# Do Monkeypox Exposures Vary by Ethnicity? Comparison of Aka and Bantu Suspected Monkeypox Cases

**DOI:** 10.4269/ajtmh.19-0457

**Published:** 2019-11-25

**Authors:** Sarah Anne J. Guagliardo, Reena H. Doshi, Mary G. Reynolds, Angelie Dzabatou-Babeaux, Nestor Ndakala, Cynthia Moses, Andrea M. McCollum, Brett W. Petersen

**Affiliations:** 1 Epidemic Intelligence Service, Centers for Disease Control and Prevention, Atlanta, Georgia;; 2 Poxvirus and Rabies Branch, Division of High-Consequence Pathogens and Pathology, National Center for Emerging and Zoonotic Infectious Diseases, Centers for Disease Control and Prevention, Atlanta, Georgia;; 3 Division of Global HIV and Tuberculosis, Center for Global Health, Centers for Disease Control and Prevention, Atlanta, Georgia;; 4 Ministry of Health, Brazzaville, Republic of Congo;; 5 Field Epidemiology Training Program, Centers for Disease Control and Prevention, Kinshasa, Democratic Republic of the Congo;; 6 International Communication and Education Fund, Kinshasa, Democratic Republic of the Congo

## Abstract

In 2017, a monkeypox outbreak occurred in Likouala Department, Republic of the Congo. Many of the affected individuals were of Aka ethnicity, hunter-gatherers indigenous to Central Africa who have worse health outcomes in comparison with other forest-dwelling peoples. To test the hypothesis that Aka people have different risk factors for monkeypox, we analyzed questionnaire data for 39 suspected cases, comparing Aka and Bantu groups. Aka people were more likely to touch animal urine/feces, find dead animals in/around the home, eat an animal that was found dead, or to have been scratched or bitten by an animal (*P* < 0.05, all variables). They were also more likely to visit the forest ≥ once/week, sleep outside, or sleep on the ground (*P* < 0.001, all variables), providing opportunities for contact with monkeypox reservoirs during the night. The Aka and possibly other vulnerable groups may warrant special attention during educational and health promotion programs.

Monkeypox predominantly affects populations residing in heavily forested regions of Central and West Africa, and recent epidemiological events suggest that its geographic range is expanding and its incidence is increasing.^1,2^ This emerging zoonosis is often found in isolated and inaccessible areas, where health-care delivery is hampered and public health surveillance is muted. People living in these isolated areas are therefore most vulnerable to monkeypox infection. The Aka people belong to the larger Bayaka ethnicity, a group of indigenous hunter-gatherers who perhaps best exemplify a vulnerable population within the Central African region. Evidence of Bayaka cultures dates back more than 20,000 years,^3^ and today, these people are threatened by warfare, logging, and the encroachment of agriculture in addition to social stigma and discrimination.^4^ Unlike the ethnic majority, the Bantu people, their nomadic lifestyle hinders access to education and health care. But even when Bayaka people abandon nomadism, they still experience worse health outcomes relative to their forest-dwelling, non-Bayaka counterparts.^5^ This raises questions as to whether Bayaka people have increased susceptibility to sylvatic zoonoses such as monkeypox as a consequence of more contact with wild animals and poor access to health services.

Clinically comparable with smallpox, monkeypox produces a febrile rash illness, including lesions on the palms of the hands and soles of the feet, with the addition of lymphadenopathy. Infection with monkeypox virus can be severe: in endemic regions, it is estimated that up to 11% of cases without prior smallpox vaccination are fatal.^6^ Monkeypox virus transmission is thought to be associated with contact with infected wild animals, yet the primary reservoir host(s) is unknown—monkeypox virus has only been isolated twice from wild animals: once from a rope squirrel (*Funisciurus anerythrus*) in the Democratic Republic of Congo (1985) and once from a sooty mangabey (*Cercocebus atys*) in Côte d’Ivoire (2012).^7,8^

In January 2017, in Likouala Department, Republic of the Congo (ROC), a large monkeypox outbreak occurred in four districts, including Impfondo, Betou, Enyelle, and Dongo.^9^ Here, we report the results of an exposure questionnaire conducted among suspected cases to test the hypothesis that the Aka people (a Bayaka group) are at greater risk of sylvatic animal exposures and therefore elevated risk of monkeypox and other zoonoses. Investigative methods used during this outbreak are described in detail elsewhere.^9^ Suspected cases were defined as persons with unexplained rash and fever (subjective or measured temperature of ≥ 99.3°F [≥ 37.4°C]) during the period January 1–April 2, 2017. Two lesion specimens and dried blood strips were collected from suspected cases; polymerase chain reaction testing was conducted at the Institut National de Recherche Biomédicale, and dried blood strips were tested at CDC for anti-Orthopoxvirus IgG and IgM antibodies by ELISA.^10^ Suspected cases were interviewed about symptoms, demographic information, and exposures to animals and other suspected monkeypox cases. Questionnaire data were captured on tablets and were imported into SAS 9.3 (SAS Institute, Cary, NC) for cleaning and analysis. The survey investigation was reviewed and given a non‐research determination by CDC’s National Center for Zoonotic and Infectious Diseases. Verbal informed consent was acquired before interviews and blood sample collection, and risks and benefits were explained in both a group setting and on an individual basis.

We analyzed complete questionnaire data for 39 suspected cases. These persons were ultimately classified as suspected, possible, probable, or confirmed cases^9^; however, transcription errors ultimately prevented us from linking complete exposure data with test results. Seven individuals ultimately met the criteria for confirmed monkeypox.^9^ For this reason, data presented here were analyzed irrespective of final case designation. We compared exposures among Aka and other ethnicities using chi-squared/Fisher’s exact tests for categorical exposures and *t*-tests or Wilcoxon rank-sum tests for quantitative variables. We assessed differences in demographics, sleeping habits that could lead to exposures (i.e., sleeping on the ground or sharing a bed), exposures to animals or other suspected cases, and finally, history and knowledge of monkeypox.

Approximately, 46% (17/39) of suspected monkeypox cases were of Aka ethnicity, and 94% (16/17) of suspected Aka cases were from Manfouété (Table 1). Only a single Aka person was identified in a different location (Betou). People from all districts were equally likely to test IgG positive, but suspected cases from Manfouété (comprising all Aka) were more likely to be IgM positive, indicating a recent exposure to an orthopoxvirus (81.3% compared with 40% in other districts, *P* = 0.013). Fishermen were more likely to be Aka and merchants were more likely to be Bantu (*P* < 0.0001). All refugees were of Bantu ethnicity (*P* < 0.05). Mean household size (8.0) and the number of children per household (4.4) were approximately equal for Aka and Bantu alike. Approximately 94% (16/17) of Aka people reported sleeping outside once during the period of interest (1 month before the investigation) (*P* < 0.0001). A similar proportion (100%, 17) of Aka suspected cases reported sleeping on the ground (*P* < 0.0001) or sleeping without a bed at least once (93.8%, 15). Aka suspected cases were also more likely to share a bed (*W* = 415.5, *P* < 0.001) and share a room (*W* = 421.5, *P* < 0.01) with more people.

**Table 1:** Characteristics of the study population

Select variable	Total (*N* = 39)	Aka (*N* = 17)	Other ethnicity (*N* = 22)	χ^2^	*P*-value
	*n* (%)	*n* (%)	*n* (%)		
Gender					
Male	11 (28.2)	6 (35.3)	5 (22.7)	0.75	0.39
Female	28 (71.8)	11 (64.7)	17 (77.3)		
Refugee					
Yes	7 (18.0)	0 (0)	7 (31.8)	NA	**0.012**
No	32 (82.1)	17 (100)	15 (68.2)		
Location					
Betou	11 (28.2)	1 (5.9)	10 (45.5)	NA	**< 0.0001**†
Enyelle	4 (10.3)	0 (0)	4 (18.2)		
Manfouété	18 (46.2)	16 (94.1)	2 (9.1)		
Impfondo	6 (15.4)	0 (0)	6 (27.3)		
Occupation					
Teacher/student	6 (15.4)	0 (0)	6 (27.3)	NA	**< 0.0001**†
Merchant	5 (12.8)	0 (0)	5 (22.7)		
Child	9 (23.1)	7 (41.2)	2 (9.1)		
Farmer	6 (15.4)	4 (23.5)	2 (9.1)		
Fisherman	5 (12.8)	3 (17.7)	2 (9.1)		
Hunter	1 (2.6)	1 (5.9)	0 (0)		
Housekeeper	3 (7.7)	0 (0)	3 (13.6)		
Other	4 (10.3)	2 (11.8)	2 (9.1)		
Frequency of sleeping outside*					
Never	18 (46.2)	1 (5.9)	17 (77.3)	NA	**< 0.0001**†
≥ Once	21 (53.9)	16 (94.1)	5 (22.7)		
Frequency of sleeping on the ground*					
Never	20 (51.3)	0 (0)	20 (90.9)	NA	**< 0.0001**†
≥ Once	19 (48.7)	17 (100)	2 (9.1)		
Frequency of sleeping without a bed					
Never	21 (56.8)	1 (6.3)	20 (95.2)	NA	**< 0.0001†**
≥ Once	16 (43.2)	15 (93.8)	1 (4.8)		
No response	2	–	–		

Bold denotes statistical significance. Column percentages are reported.

* During the time period of interest (1 month before the investigation).

† Fisher’s exact test *P*-value.

Analysis of exposures (Table 2) showed that Aka people were more likely to venture into the forest ≥ once per week (*P* < 0.0005). Aka suspected cases were also more likely to touch urine or feces of animals (*P* < 0.0001), be bitten (*P* < 0.05) or scratched by animals (*P* < 0.005). The Aka people were more likely to have found (*P* < 0.05) and eaten an animal that was found dead (*P* < 0.005). Contact with another person with rash illness during the period of interest was reported by more Aka than Bantu people (100% versus 63.5%, respectively, *P* < 0.01). Aka people were less likely to report a prior history of monkeypox (*P* < 0.001) and more likely to report the occurrence of a similar rash illness in the community (*P* < 0.05) (Table 3).

**Table 2:** Comparison of exposures

Select variable	Total (*N* = 39)	Aka (*N* = 17)	Other ethnicity (*N* = 22)	*P*-value†
	*n* (%)	*n* (%)	*n* (%)	
Forest visitation frequency				
Never	19 (54.3)	2 (14.3)	17 (81.0)	**0.0001**
≥ Once	16 (45.7)	12 (85.7)	4 (19.0)	
No response	4	–	–	
Dead animal found inside/near home*	4 (10.8)	3 (20)	1 (4.6)	**0.014**
No	33 (89.2)	12 (80)	21 (95.5)	
No response	2	–	–	
Touched animal urine/feces*	11 (33.3)	11 (78.6)	0 (0)	**< 0.0001**
No	22 (66.7)	3 (21.4)	19 (100)	
No response	6	–	–	
Scratched by an animal*	6 (16.7)	6 (40)	0 (0)	**0.0026**
No	30 (83.3)	9 (60)	21 (100)	
No response	3	–	–	
Bitten by an animal*	7 (19.4)	6 (40)	1 (4.8)	**0.013**
No	29 (80.6)	9 (60)	20 (95.2)	
No response	3	–	–	
Ate an animal that was found dead*	6 (15.8)	6 (35.3)	0 (0)	**0.0045**
No	32 (84.2)	11 (64.7)	21 (100)	
No response	1	–	–	
Contact with another person with rash*	31 (79.5)	17 (100)	14 (63.6)	**0.0056**
No	8 (20.5)	0 (0)	8 (36.4)	

Bold denotes statistical significance. Column percentages are reported.

* During the time period of interest (1 month before the investigation).

† Fisher’s exact test *P*-value.

**Table 3:** Comparisons of history and knowledge of monkeypox

Select variable	Total (*N* = 39)	Aka (*N* = 17)	Other ethnicity (*N* = 22)	*P*-value†
	*n* (%)	*n* (%)	*n* (%)	
History of monkeypox	11 (28.2)	0 (0)	11 (50)	**0.0007**
No	28 (71.8)	17 (100)	11 (50)	
Prior knowledge of monkeypox	28 (73.7)	14 (87.5)	14 (63.6)	0.14
No	10 (26.3)	2 (12.5)	8 (36.4)	
No response	1	–	–	
Occurrence of similar rash illness in village	27 (69.2)	15 (88.2)	12 (54.6)	**0.037**
No	12 (30.8)	2 (11.8)	10 (45.5)	
Occurrence of similar rash illness in home	27 (69.2)	14 (82.4)	13 (59.1)	0.17
	12 (30.8)	3 (17.7)	9 (40.9)	

Bold denotes statistical significance. Column percentages are reported.

* During the time period of interest (1 month before the investigation).

† Fisher’s exact test *P*-value.

Our results show that Aka people with suspected monkeypox are more likely to exhibit several important risk factors, including frequently venturing into the forest, virtually all types of contact with animals, and sleeping outside and on the ground, which in turn may increase the probability of animal contact during the night. This leads us to propose that Aka people are more likely to be exposed to sylvatic animals, and are therefore at greater risk of acquiring zoonotic infection. Relatedly, Bantu and Aka groups could exhibit different “exposure profiles,” or combinations of behavioral and situational risk factors related to monkeypox and other zoonoses.

Previous research is supportive of the notion that Aka ethnicity is associated with elevated risk of monkeypox: a serosurvey carried out in Likouala Department found a higher occurrence of anti-Orthopoxvirus IgM antibody among Aka compared with Bantu populations (7.3% versus 1.5%, respectively, *P* = 0.005).^11^ Further investigation is required to understand the exposure profiles of different populations and subgroups of people (e.g., ethnic groups and age-groups). Past research in the Congo Basin has revealed differing hunting patterns between Bayaka and other ethnic groups,^3^ but further research is required to determine whether these differences might be related to exposure to possible monkeypox reservoirs.

Our findings are relevant for the control and prevention of zoonotic diseases. Ebola, for instance, may serve as a parallel system to monkeypox, as its transmission is similarly driven by contact with wild animals. Previous works have demonstrated a significantly higher seroprevalence of Ebola virus in Bayaka versus non-Bayaka in the Central African Republic,^12^ and an overall high rate of Ebola seropositivity in the Democratic Republic of the Congo.^13^

Our results should be interpreted with some discretion, as the data from 2017 Likouala outbreak were not collected for the explicit purpose of comparing different exposure types by ethnic groups. The sample population in this analysis is not random, and therefore the behaviors and demographics of those impacted by the outbreak are not representative of the broader population. The investigation team focused on case identification, but other cases, particularly those with more mild forms of the disease could have been missed. Furthermore, in the absence of baseline census data, it is unclear whether Aka people in Manfouété were disproportionately affected by rash illness or, alternatively, whether this group is simply more prevalent in Manfouété (sampling bias). Last, with just 39 suspected cases, the sample size in this investigation is small, limiting our ability to conduct more robust statistical modeling.

We postulate that Bayaka populations are at greater risk of sylvatic zoonoses than the general population as they frequently report risk factors for monkeypox, such as hunting and butchering bush meat and frequent contact with wildlife. It is estimated that the Bayaka represent upward of 900,000 people in the Central African rainforests,^14^ yet little attention has been paid to this neglected group and their risk of zoonoses. Additional research is warranted and targeted educational and prevention efforts could be beneficial to this population.
